# Deep brain electrical neurofeedback allows Parkinson patients to control pathological oscillations and quicken movements

**DOI:** 10.1038/s41598-021-87031-2

**Published:** 2021-04-12

**Authors:** Oliver Bichsel, Lennart H. Stieglitz, Markus F. Oertel, Christian R. Baumann, Roger Gassert, Lukas L. Imbach

**Affiliations:** 1 Rehabilitation Engineering Laboratory, Department of Health Sciences and Technology, ETH Zurich, Zurich, Switzerland; 2 Department of Neurology, University Hospital Zurich, University of Zurich, Zurich, Switzerland; 3 Department of Neurosurgery, University Hospital Zurich, University of Zurich, Zurich, Switzerland; 4 Clinical Neuroscience Centre, University Hospital Zurich, University of Zurich, Zurich, Switzerland; 5 Swiss Epilepsy Center, Klinik Lengg, Zurich, Switzerland

**Keywords:** Neuroscience, Neurology

## Abstract

Parkinsonian motor symptoms are linked to pathologically increased beta-oscillations in the basal ganglia. While pharmacological treatment and deep brain stimulation (DBS) reduce these pathological oscillations concomitantly with improving motor performance, we set out to explore neurofeedback as an *endogenous* modulatory method. We implemented real-time processing of pathological subthalamic beta oscillations through implanted DBS electrodes to provide deep brain electrical neurofeedback. Patients volitionally controlled ongoing beta-oscillatory activity by visual neurofeedback within minutes of training. During a single one-hour training session, the reduction of beta-oscillatory activity became gradually stronger and we observed improved motor performance. Lastly, endogenous control over deep brain activity was possible even after removing visual neurofeedback, suggesting that neurofeedback-acquired strategies were retained in the short-term. Moreover, we observed motor improvement when the learnt mental strategies were applied 2 days later without neurofeedback. Further training of deep brain neurofeedback might provide therapeutic benefits for Parkinson patients by improving symptom control using strategies optimized through neurofeedback.

## Introduction

Patients with Parkinson’s disease (PD) suffer from progressive impairment of motor function caused by a loss of dopaminergic neurones in the basal ganglia and brainstem^1^. Symptomatic treatments of PD include dopaminergic drugs or deep brain stimulation (DBS). In DBS, electrodes are implanted into basal ganglia nuclei—typically the subthalamic nucleus (STN) or the globus pallidus internus (GPi)—both of which are relevant structures in affected movement modulatory circuits^2^. Besides greatly and almost immediately alleviating motor symptoms through the application of high frequency stimulation, implanted DBS electrodes have also shed light on the electrophysiological hallmarks of PD through recording of deep brain local field potentials (LFP).

Characteristically, patients with PD show pathologically increased levels of beta-oscillatory activity^3–5^. Beta-oscillations have been correlated with clinical measures of PD, with a reduction of signal power in the beta-band corresponding to a clinical improvement of motor symptoms^6–8^. Furthermore, the two main therapeutic strategies, the administration of l-Dopa^3,9^ and high-frequency DBS^8,10^, both lead to a suppression of beta-synchronicity in the STN. Beta-oscillations show fast and movement-dependent modulation over time^11,12^ and have been proposed as a feedback signal to control the delivery of DBS in a closed-loop system^13^.

Besides the *exogenous* manipulation of subthalamic oscillatory activity through pharmacotherapy and DBS, it remains to be unveiled whether *endogenous* modulation is also achievable. Neurofeedback has emerged as a promising, *endogenous* technique enabling self-regulation of ongoing brain activity. It relies on the real-time extraction of relevant features from neuronal activity, that is presented to the subject. Such a real-time self-modulation would be especially useful in sudden exacerbations of symptoms, which are characteristic for PD. *Cortical* modulation of oscillations through electrical neurofeedback has already been shown successful in a sample of three PD patients controlling beta-band power from sensorimotor areas^14^, as well as in three macaques where the downregulation of cortical beta-activity resulted in significantly faster movement onsets^15^.

The modulation of *deep brain* activity through neurofeedback is not a new notion per se and has been explored by a large body of real-time functional magnetic resonance imaging (rtfMRI) research, providing for instance evidence for voluntary control over the substantia nigra/ventral tegmental area complex (SN/VTA) through mental imagery^16^. However, compared to LFP recordings, rtfMRI is confined to the laboratory environment, suffers from a time lag of several seconds caused by the haemodynamic response and is limited by a low temporal resolution of typically 1–2 s. Using LFP recordings from DBS electrodes, a recent study showed that the 5 min average resting subthalamic beta-activity could be voluntarily controlled in the instructed direction through 10 min of neurofeedback^17^. Nevertheless, it remains to be unveiled whether deep brain electrode-guided neurofeedback allows for instantaneous modulation of ongoing subcortical activity as well as whether modulatory effects improve with exposure time, are maintained after removal of neurofeedback and reduce motor impairment.

We hypothesise that through electrical deep brain neurofeedback, PD patients can learn to volitionally control the amount of pathological subthalamic beta-oscillations in *real-time* and thereby reduce motor impairment. Ultimately, *endogenously* modulating pathological deep brain oscillations could serve as a new approach to reduce Parkinsonian motor symptoms.

## Materials and methods

### Study cohort and surgery

We included ten PD patients (Table 1) undergoing bilateral DBS lead (Model 3389, Medtronic Neurological Division, Minneapolis, MN, USA) implantation into the STN. DBS leads were implanted after MRI-based direct targeting of the STN. Accurate implantation within the STN was intraoperatively verified by micro-electrode recordings (Leadpoint, Medtronic Neurological Division, Minneapolis, MN, USA) in steps of 0.5 mm until the pars reticulata of the substantia nigra (starting 10 mm above the target), as well as clinical response upon intraoperative stimulation and intraoperative computed tomography (CT). We verified accurate electrode placement by comparing the planned and actual electrode position in the postoperative CT scan. As commonly performed at our institution, the internalisation and connection of the leads to the stimulation device (Activa PC, Medtronic Neurological Division, Minneapolis, MN, USA) was performed 3–5 days after the first surgery (two-stage surgical approach). All experiments were performed with externalised DBS leads on the second postoperative day in l-Dopa OFF as well as DBS OFF state and prior to the second stage of surgery. Decisions on patient selection and surgical procedures were not affected by this study and exclusively based on clinical grounds. This study was approved by the local ethics committee (Kantonale Ethikkommission Zürich, board decision on the amendment to KEK-ZH: 2012–0327), and all patients gave informed written consent prior to inclusion.

**Table 1:** Demographics and clinical patient characteristics.

ID	Age (years)	Gender	PD type	Side dominance	Disease duration (years)	Hoehn-Yahr Scale	MDS-UPDRS III ON/OFF	LED (mg/day)
1	70	M	Equivalent	Left	10	2.5	37/62	2340
2	63	M	Akinetic-rigid	Right	7	2.5	27/44	1800
3	45	M	Tremor	Right	2	1.5	19/28	375
4	60	M	Tremor	Right	19	1.5	17/41	1750
5	62	F	Akinetic-rigid	Right	10	2.5	29/46	275
6	65	M	Equivalent	Right	9	2	20/32	1240
7	57	F	Equivalent	Right	9	2	25/54	620
8	71	M	Tremor	Left	4	2.5	19/35	1350
9	58	F	Equivalent	Right	13	2.5	20/33	1500
10	48	M	Equivalent	Left	5	2	24/43	1525

The third part of the MDS-UPDRS includes the clinician-scored, monitored motor evaluation (33 scores based on 18 items)^19^, ranging from the worst MDS-UPDRS III score of 132 (= 4.33) points to 0 points (as in healthy individuals). The minimal clinically important difference on the UPDRS motor score is 2.3–2.7 points^20^. For patients 1, 8 and 10, the PD-dominant hand was the left hand, while for all others the PD-dominant hand was the right hand.

*MDS-UPDRS* Movement Disorder Society-Unified Parkinson's Disease Rating Scale, *ON/OFF* values of preoperative l-Dopa challenge test, *LED* preoperative levodopa equivalent dose^18^.

### Experimental setup

We measured STN LFPs from temporarily externalised DBS wires (4 electrode sites per lead) sampled at 5 kHz by a BrainAmp DC (Brain Products GmbH, Gilching, Germany) EEG recorder. A ground and a reference scalp EEG input were placed over the central midline region C_z_ (according to the international 10/20 system) and connected to the EEG recorder. All electrode impedances were < 5 kOhm and signal quality was checked visually for electrode or movement artefacts. All experiments were performed in a sitting or supine position such that patients were able to see the computer screen providing visual feedback. As a baseline condition, we first recorded a resting state condition in which patients looked at a blacked monitor for 60 s. Using the BrainVision Recorder and Analyzer (both from BrainProducts GmbH, Gilching, Germany), we then identified the pair of adjacent electrode contacts in the STN contralateral to the PD-dominant hand that showed the highest beta power in the power spectral density and determined the beta-peak frequency as an individual parameter to be used for signal processing^11^. For patients 1, 8 and 10, the PD-dominant hand was the left, while for all others the PD-dominant hand was the right.

### Visual neurofeedback

**Figure 1:**
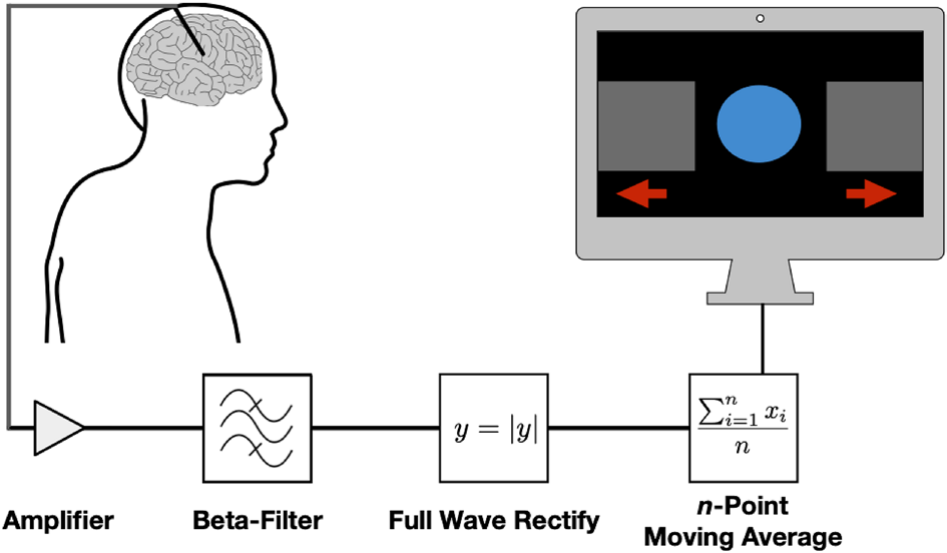
Deep brain electrical neurofeedback loop. Deep brain stimulation electrodes (upper left corner) implanted into the subthalamic nucleus allowed for brain oscillation measurements. The raw, analogue signal was amplified and then digitally band-pass filtered around the individual beta-peak (± 5 Hz), rectified, averaged and used to control the position of the blue disc on the monitor (upper right corner). Patients tried to volitionally control the position of the disc, thereby closing the neurofeedback loop while controlling pathological beta-activity.

We implemented a custom-written C++-programme to extract an instantaneous beta-power estimate from the externalised leads. The programme built on the BrainAmp Software Development Kit (Version 002, BrainProducts GmbH, Gilching, Germany), which provided a framework allowing direct access to the BrainAmp EEG amplifier through a USB port. Every 40 ms, 200 samples (0.04 s * 5 kHz) per channel were bussed through the USB port for processing of the raw signal: First, a bipolar channel was created by subtracting one channel from its adjacent channel; second, the raw bipolar lead signal was band-pass filtered (Butterworth, 6th order) around the previously determined individual beta peak-frequency ($\pm$ 5 Hz) by a rational transfer function; third, the signal was rectified; fourth, the signal from this and the previous 4 blocks was averaged (running window average). Finally, the resulting value, representing the instantaneous smoothed beta-activity over the preceding 200 ms, was updated at a rate of 25 Hz and used for visual neurofeedback. This real-time neurofeedback parameter determined the location of a disc that moved horizontally from lower values on the left of the screen to higher values on the right (Fig. 1). The lower limit of the screen was defined as the 75th percentile beta-power value obtained during 1 min of overt fist opening and closing with the PD-dominant hand. The upper limit of the screen was defined as the 75th percentile neurofeedback value during 1 min of staring at the blacked computer screen.

### Neurofeedback experiment

The experiment was divided into three parts comprising several 1-min blocks: the pre-neurofeedback, the neurofeedback and the transfer part (Supplementary Fig. 1). During the pre-neurofeedback part, patients had no prior neurofeedback training and were given strategies that might help to up- or downregulate beta-activity. It consisted of one block of baseline (staring at the blacked computer screen), followed by downregulation, where patients were instructed to imagine fluent movements; and upregulation, where patients were instructed to imagine bradykinetic or abruptly ending movements. For the neurofeedback (NF) rounds, we provided visual neurofeedback for all blocks except for the baseline blocks. During neurofeedback, patients were instructed to voluntarily move the disc on the screen to the right side of the screen (upregulation) or to the left of the screen (downregulation) without overt movements (visually controlled by the examiner). Patients were allowed to adapt their mental strategies based on success. The following sequence of blocks was repeated three times (NF1, NF2 and NF3; except for Patient 1, in which NF2 had to be omitted due to time restrictions): baseline, downregulation, upregulation, baseline, upregulation, downregulation. The NF 3 sequence was repeated for the transfer round, where the computer screen was again turned off (no neurofeedback) and patients were instructed to reuse the previously learnt mental strategies for either up- or downregulating beta-activity while staring at the black computer screen and avoiding movements. In total, each patient underwent 30 blocks (24 blocks for patient 1) resulting in a raw experimental time of 30 min (24 min for patient 1). Allowing for short transition periods between the blocks, the total experimental time was usually between 40 and 50 min. Moreover, we noted the final mental strategies used by the patients to either up- or downregulate during the last round of neurofeedback (NF3) for later analysis.

To capture the behavioural effects of neurofeedback, each block during NF3 was immediately followed by a period of 15 s, where patients were instructed to alternatingly and completely (i. e. 180° rotations) pro- and supinate their more severely affected hand as fast as possible. We used an inertial measurement unit (IMU) mounted on the hand to log the recordings at 200 Hz (ZurichMOVE, https://www.zurichmove.com). Two days after the initial neurofeedback experiment (i. e. after DBS lead internalisation and thus without neurofeedback), we assessed the effect of neurofeedback-learnt mental strategies on motor performance (again in L-DOPA OFF and DBS OFF) during a repetition of the transfer round with the pro- and supination motor task following each block.

Since the bidirectional neurofeedback design allowed for intra-individual control, differences in the amount of beta-oscillations would be solely attributable to the neurofeedback condition, making a separate control group superfluous. Moreover, the bidirectional neurofeedback design also controlled for possible eye movement contributions to neuromodulation. The study was single-blinded, i. e. patients were unaware of the neuromodulatory effect (beta-upregulation vs. downregulation) of moving the disc to either side.

### Post-hoc data analysis and statistics

The raw signal from each electrode site was saved in the BrainAmp EEG format and later converted to European Data Format (edf) by BrainVision Analyzer (BrainProducts GmbH, Gilching, Germany). The edf was imported into MATLAB (R2018b, The MathWorks Inc., Natick, Massachusetts, USA) for all further data analyses and statistics. To reconstruct the previously visualised signals, we calculated the same bipolar montage from the raw data and filtered using the same algorithms as during the real-time programme. The signal from each block was later rectified and averaged. The averaged values obtained for the up- or downregulation blocks were then normalised with the values from the preceding baseline block. Finally, the normalised values from the same condition of the same block (pre-neurofeedback, NF1, NF2, NF3, transfer) were averaged for between condition comparison. Hypothesis testing with two-sided Student’s *t*-test was used to determine whether neurofeedback significantly modulated the instantaneous beta-power. We used Wilcoxon’s signed rank test to compare the pre-neurofeedback with the transfer values. Additionally, we used R (version 4.0.2)^21^ and lme4^22^ to perform linear mixed effects analyses of the relationship between the beta-power estimate and the neurofeedback time as well as neurofeedback direction. For all linear mixed effect models, we had subject as a random intercept and random slope effect and calculated *p*-values by likelihood ratio tests with the effect in question against the model without the effect in question. In order to assess the effect of neurofeedback downregulation time on the beta-power estimate, we tested the full model with the fixed effect of neurofeedback time against the model without the fixed effect of neurofeedback time. For effects in the bidirectional neurofeedback design, we tested the full model with the fixed effects of neurofeedback time and neurofeedback direction against the model without the fixed effect of neurofeedback direction. We correlated the MDS-UPDRS III OFF score with the beta-modulatory capacity to assess the relationship between disease burden and the ability to modulate pathological oscillations, i. e. the maximum percent difference between the average beta-power during the baseline rest block (100%) and its subsequent downregulation block during the neurofeedback part. The gyroscope data from the IMU was imported into MATLAB. A custom-written script summed the three pairwise-orthogonal gyroscope axes up before integration and band-pass filtering between 0.25 and 4 Hz (6^th^ order Butterworth). To examine the motor performance, the pro- and supination segments were visually identified for each patient and truncated to the first 12 s (informed decision to avoid the effect of prematurely terminated movements). The following metrics were calculated and averaged over repetitions of the same condition for each individual patient: number of pronosupination cycles (measure of frequency), mean peak angular amplitude (measure of movement extent), cumulative angular displacement (measure of total movement extent) and mean absolute angular acceleration (measure of the torque generated by the antagonistically acting pro- and supinator muscles). We used Wilcoxon’s signed rank test to compare the behavioural metrics after rest versus downregulation.

### Human participants statement

This research project involving human participants has been approved by the local ethics committee (Kantonale Ethikkommission Zürich, board decision on the amendment to KEK-ZH: 2012-0327), and all patients gave informed written consent prior to inclusion. This research project was performed in accordance with the Declaration of Helsinki.

## Results

From the 10 patients included in this study, we had to exclude two patients because of intermittent high amplitude artefacts in the raw DBS signal, resulting in a total number of 8 patients included for data analysis. The contact pairs for the bipolar recording were: 1 × C0–C1, 5 × C1–C2, 1 × C2–C3 and 3 × C9–C10. The mean ($\pm$ standard deviation) beta-peak frequency was 20.7 $\pm$ 3.3 Hz.

### Beta-activity estimate during downregulation vs. rest

**Figure 2:**
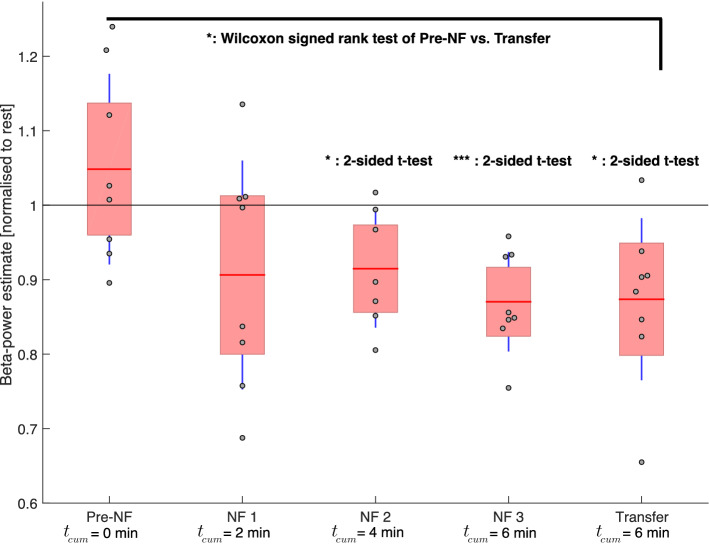
Learning to downregulate beta activity with DBS-neurofeedback. Beta-power estimates from all patients during downregulation—normalised to the beta-power estimate during each patients’ preceding rest block—are shown for the baseline (pre-NF), neurofeedback (NF1, NF2, NF3) and transfer rounds. We indicate the cumulative amount of time *t*_*cum*_ that patients have spent learning downregulation through neurofeedback until the end of that respective round. The group means are represented by the horizontal red lines, the standard deviations by the vertical blue lines and the 95% confidence intervals by the red patched areas. We used the two-sided Student’s *t*-tests to test for significant beta-reductions compared to the baseline rest (horizontal black line at 1). Wilcoxon’s signed rank test was used to compare the dependent samples from the transfer round with their pre-NF value. *p < 0.05; ***p < 0.001.

**Figure 3:**
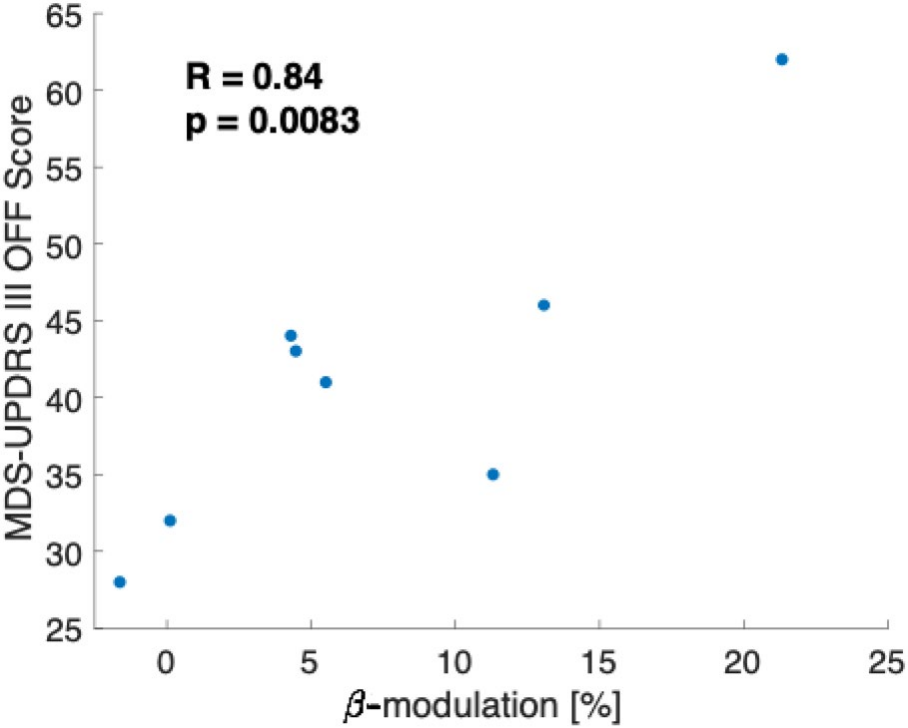
Relationship between disease burden and beta-modulatory capacity. We correlated the MDS-UPDRS III OFF score with the beta-modulatory capacity of each patient (*n* = 8) and found a significant relationship (*p* = 0.0083) with a positive Pearson correlation coefficient (*R* = 0.84).

We calculated average beta-activity estimates normalised by the beta-activity estimate during the preceding rest block for each individual patient during downregulation in pre-NF, all active neurofeedback rounds (NF1, NF2, NF3) and the transfer round (Fig. 2, sample recording in Supplementary Fig. 2). The sample mean is below 1 for all but the pre-NF round, indicating that neurofeedback resulted in reduced beta-activity compared to baseline and was significant (Student’s *t*-test) for NF2 (*p* = 0.0294), NF3 (*p* = 0.00092) and the transfer round (*p* = 0.0134). The beta-activity estimates for the transfer round were also significantly lower than during the pre-NF round (Wilcoxon’s signed rank test, *p* = 0.0156). The variance decreased from NF1 up to NF3 and again increased for the transfer round. In the linear mixed effects model analysis, neurofeedback affected the beta-power estimate (${\chi }^{2}=7.4114, p=0.006481$), lowering it by about 5.2% (relative to rest) per 2 min of neurofeedback training. More pronounced beta-modulations were achieved by patients with heavier disease burden, as represented by the MDS-UPDRS III OFF score (Fig. 3, significant relationship: *p* = 0.0083; Pearson correlation coefficient: *R* = *0*.84).

### Beta-activity estimate during upregulation vs. downregulation

**Figure 4:**
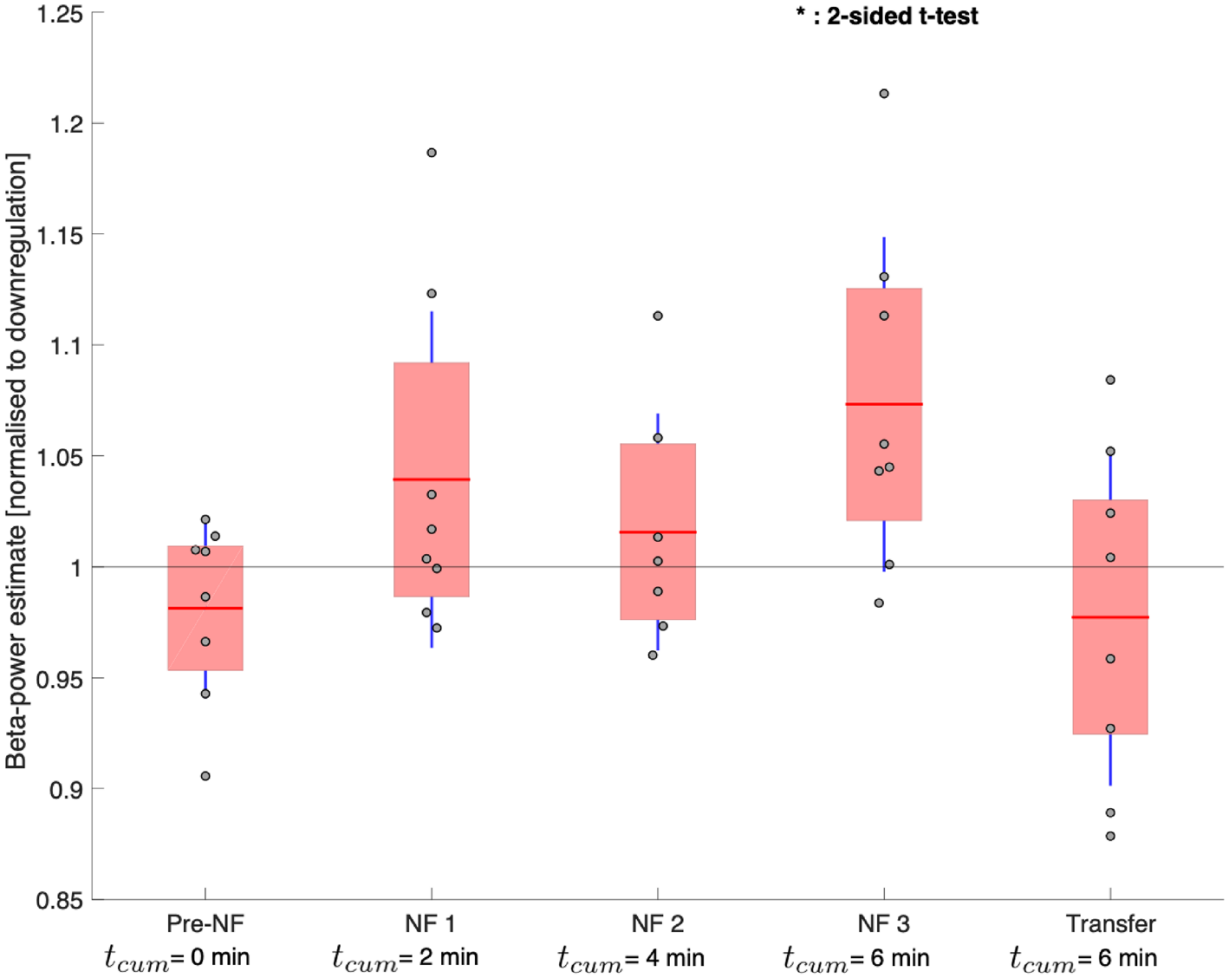
Learning bidirectional neurofeedback. Beta-power estimates from all patients during upregulation—normalised to the beta-power estimate during each patients’ downregulation condition—for the Pre-NF, NF1, NF2, NF3 and Transfer rounds show increasing beta activity upon neurofeedback aided upregulation. We indicate the cumulative amount of time *t*_*cum*_ that patients spent learning upregulation through neurofeedback until the end of that respective round. The means are represented by the horizontal red lines, the standard deviations by the vertical blue lines and the 95% confidence intervals by the red patched areas. We used two-sided Student’s *t*-testing to determine significant beta-increases compared to the downregulation condition (horizontal black line at 1). *p < 0.05.

The average beta-activity estimates during upregulation in the pre-NF, NF1, NF2, NF3 and the transfer round were calculated and normalised by the beta-activity estimate during downregulation of the same round (Fig. 4). The sample means were above 1 for all neurofeedback rounds, meaning that the beta-activity estimates were increased as compared to downregulation. For NF3, the sample mean was significantly increased in Student’s *t*-testing (*p* = 0.0288). In the linear mixed effects model analysis, neurofeedback direction was a significant fixed effect (${\chi }^{2}=7.2333, p=0.007156$).

### Mental strategies used

The final mental strategies used for up- or downregulating beta-activity were all different from the initially suggested ones and highly personalised: Strategies for downregulation ranged from imagining running, walking or cycling to just ‘moving the disc to the left’; Strategies for upregulation included imagining abrupt movements, difficulties in initiating a movement, freezing, freezing during catching a fish, relaxation and trying to ‘move the disc to the right’.

### Behavioural outcome after neurofeedback and short-term retention of mental strategy

**Figure 5:**
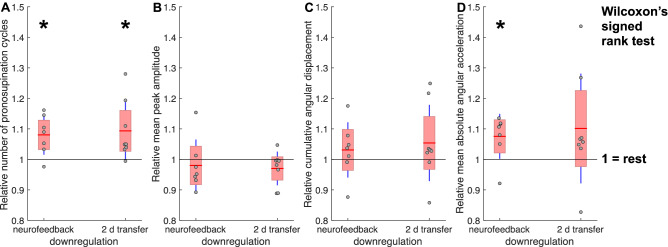
Behavioural output metrics during the first 12 s of pronosupination after downregulation during NF3 and short-term (2 days) mental strategy transfer. **(A)** The pronosupination frequency (proportional to the number of cycles during the first 12 s of pronosupination) was significantly increased after downregulation as compared to rest (*p* = 0.0312 and *p* = 0.0156 during NF3 and 2 d mental strategy transfer, respectively) while **(B)** the mean peak angular amplitude was only slightly and non-significantly decreased. **(C)** The cumulative angular displacement (i. e. degrees travelled) was generally higher after downregulation as compared to rest. The grey rectangle highlights the mutual dependence between the three variables in **(A–C)**, i. e. frequency * mean peak angular amplitude = cumulative angular displacement. **(D)** The torque generated by the antagonistically acting pro- and supinator muscles was estimated through the mean absolute angular acceleration, which was significantly increased after downregulation as compared to rest during the neurofeedback experiment (*p* = 0.0469).

To test for a behavioural effect of neurofeedback-learnt mental downregulation strategies, a set of movement metrics for the first 12 s of pronosupination (sample motor behaviour in Supplementary Fig. 3) during the rest condition was compared with the downregulation condition in 7 of the 8 patients (failure of recording in 1 patient). The movement frequency was significantly improved (Wilcoxon’s signed rank test, *p* = 0.0312) after downregulation (Fig. 5A left). To test for a bias towards smaller movement amplitudes, we calculated the mean peak angular amplitude, which was slightly and non-significantly reduced (Fig. 5B left). The cumulative angular displacement, a combination of the movement frequency and the mean peak angular amplitude, still favoured the downregulation condition (Fig. 5C left). Furthermore, the mean angular acceleration was significantly increased after downregulation as compared to the rest condition (Wilcoxon’s signed rank test, *p* = 0.0469), indicating a significantly higher mean torque generated after downregulation (Fig. 5D left). We also examined the motor performance after application of neurofeedback-learnt and retained mental strategies in all 8 patients after 2 days, when DBS leads had already been internalised and neurofeedback could no longer be provided, and found a similar pattern (Fig. 5; Wilcoxon’s signed rank test for the number of pronosupination cycles, *p* = 0.0156).

## Discussion

Many studies have provided evidence for a mutual interaction between beta-oscillatory activity in the STN and motor symptoms in PD. Inspired by real-time fMRI studies providing proof-of-principle for endogenous modulation of deep brain networks (although cumbersome and not transferable into everyday life), we investigated a novel neurofeedback technique using DBS electrodes implanted in the STN. Using this approach, we provided real-time direct visual neurofeedback of subcortical beta-power in the form of a disc that moved horizontally on a screen, thereby empowering patients to control their own pathological deep brain oscillations. The core findings of this study were that deep brain electrode-guided neurofeedback allows PD patients to gain control over subthalamic beta-oscillations, that this control is gradually improved with neurofeedback training and has a positive effect on motor performance. Furthermore, our findings suggest that neurofeedback-learnt strategies can be retained in the immediate and short-term and that the associated beta-power reduction has a beneficial effect on motor performance observed 2 days after neurofeedback-learning.

### Deep brain electrical neurofeedback quickly enables control over deep brain activity

Here, we demonstrate that real-time modulation of beta-activity is possible, even within as little as 4 min of downregulation training (*p* = 0.0294) and 6 min of upregulation training (*p* = 0.0288). This minimal-latency, endogenous access to deep brain activity greatly outperforms rtfMRI neurofeedback studies, where the control over the BOLD signal requires at least 30 min of training. In comparison to EEG neurofeedback studies, that require multiple sessions for controlling cortical activity^23^, DBS neurofeedback seems to provide faster and more reliable strategy development for the participants. This striking difference could be explained by local measurement of neuronal activity directly at the target deep brain area of basal ganglia-cortical loops^11,24^. More so, electrical neurofeedback of the STN seems to be readily achievable, as all patients in our study managed to reduce pathological oscillations after only 6 min of downregulation training. Importantly, given our bidirectional neurofeedback design (within-subject control strategy, i. e. all patients serve as their own control), the modulatory effect can be attributed to the learning process of neurofeedback.

### Control over deep brain activity rapidly improves with further neurofeedback-training

We demonstrate that lengthening the exposure to visual neurofeedback results in a better control over pathological beta-oscillations, as shown in a stronger and more significant reduction of beta-oscillations by the end of NF 3 (Fig. 2). Since one individual achieved a beta-reduction of $\sim$ 35% and we did not see any plateau effect in the learning curve by the end of 8 min of downregulation training, we hypothesise that stronger beta-reductions could yet be achieved through longer training sessions. Upregulation through neurofeedback was less effective (not-significant compared to rest), which might be due to the reason that beta-activity during rest is known to be pathologically increased in PD and resting activity was used as baseline condition. Nevertheless, upregulation through neurofeedback was an important within-subject control condition to prove bidirectional neurofeedback control, where factors such as concentration, visual feedback and eye movements were nearly identical and could thus be excluded as potential confounders. Of note, the initial strategy to downregulate (pre-NF) on average lead to a higher beta-activity as compared to the resting state. This suggests that controlling deep-brain activity without neurofeedback does not seem to be intuitive. Moreover, neurofeedback helps in selecting and optimising mental strategies, as all patients reported a different strategy by NF3.

### After training, beta-oscillations can be reduced without neurofeedback

Even in the absence of visual neurofeedback and after only 6 min of effective downregulation learning, PD patients still managed to significantly reduce subthalamic beta-activity using their novel neurofeedback-learnt strategies. Performance during this condition (transfer run) was also significantly better than before neurofeedback learning (pre-neurofeedback run) as evaluated by the Wilcoxon’s signed rank test. We observed an increase of variance in the amount of beta power downregulation from NF 3 to the transfer round. We believe that this difference is due to the fact that visual neurofeedback is no longer provided and that some subjects manage to obtain even stronger neuromodulation (possibly because they could concentrate better on the learned mental strategy) while some subjects perform worse, possibly due to the missing visual cues. Overall, we observed a slightly decreased downregulation ability without visual neurofeedback. Thus, continuous neurofeedback, besides allowing for further learning, also allows for a more efficient control over deep brain activity.

### Motor performance improvements

The analysis of motor performance after employing neurofeedback-learnt strategies revealed promising results in behavioural outcome metrics. Immediately after neurofeedback-guided beta downregulation, the pronosupination frequency was significantly higher while movement amplitude was only slightly and non-significantly reduced, still resulting in a larger cumulative angular displacement. Furthermore, the mean torque generated, estimated by the mean absolute angular acceleration and as such a measure for alternating activation of pro- and supinator muscles of the forearm, was significantly increased after downregulation. A similar pattern was observed 2 days later, suggesting that neurofeedback-learnt mental strategies are retained in the short-term and motor performance improvements can be achieved without active neurofeedback. A limitation of this study is that we only included a single motor task instead of adding also a second task, for instance from the MDS-UPDRS. As such, we also only focused determined the effect of neurofeedback on bradykinesia and were blind to effects on for instance tremor or non-motor symptoms. Furthermore, we assessed the effect of neurofeedback-learnt mental strategies on a single time point (i. e. 2 days) after neurofeedback learning since at that point we expected all patients to still be at the hospital and not at a remote rehabilitation clinic and as we expected a better comparability of behavioural performance (a long-term time point might have shown overall better baseline motor performance).

### Implications for Parkinson’s disease and beyond

We showed that self-control over pathological deep brain oscillations can be achieved through DBS-neurofeedback, which in turn improved motor performance in our cohort. Given the strongly fluctuating nature of PD, neurofeedback-learnt strategies to control pathological deep brain oscillations could help patients cope with situations of transient symptomatic exacerbations: PD patients could apply neurofeedback-learnt strategies to overcome sudden symptomatic exacerbations. Interestingly, the beta-modulatory capacity correlates with disease burden (Fig. 3), possibly reflecting that a pronounced pathological background beta-activity is required for stronger beta-modulation. This finding further motivates the use of deep-brain neurofeedback especially during symptom exacerbations or in severely affected patients. Although our results have to be interpreted cautiously, we provide evidence that endogenous modulation of pathological beta activity may result in improved motor control and thus complement dopaminergic treatment or brain stimulation. Thus, neurofeedback could also result in a reduced overall exposure to medication and brain stimulation, thereby combating the development of tolerance as well as potentially having a halting or reversing effect on the natural progression of PD through neuroplasticity by repeated self-activation of neuronal circuits. Along this line, a recent study was able to show that neurofeedback of deep brain beta-activity induced changes in the resting oscillatory activity before versus after neurofeedback in the respective direction of neurofeedback^17^.

These effects can be expected to be more pronounced when longer neurofeedback training is provided using DBS systems that wirelessly transmit signals to an external device^25^ for real-time neurofeedback. Finally, neurofeedback can reincorporate PD patients into the treatment loop: Compared with current DBS strategies, where PD patients are mere bystanders with little to no control over their burden (and therapy), PD patients could exert neurofeedback-learnt control over pathological deep brain activity in order to reduce stimulation and medication load while simultaneously increasing motor performance through endogenous beta reduction. As DBS has recently also been explored for neurological diseases like epilepsy, obsessive–compulsive disorder, anxiety, depression and attention deficit/hyperactivity disorder, deep brain electrode-guided neurofeedback could be simultaneously studied as a complementing treatment strategy. We showed that not only unidirectional but also bidirectional neurofeedback control can be achieved within a matter of minutes, thus making neurofeedback interesting for cases where a stabilisation or reinforcement of oscillatory patterns is beneficial.

### Outlook

The full extent to which voluntary self-regulation of deep brain oscillatory activity is possible and improves motor outcome yet remains to be unveiled as our study was limited by a short intervention time. We believe that Parkinsonian motor symptoms can be improved by providing longer neurofeedback intervention times, ideally chronic neurofeedback in the context of an implantable device. Improved motor control promoted through these optimised neurofeedback interventions will need to be selectively verified in activities of daily living. Furthermore, future studies should investigate combinatorial approaches and assess whether neurofeedback can reduce the dependence on dopaminergic medication or electrical stimulation, thereby possibly reducing medication side effects or prolonging battery life, respectively.

## Conclusion

This intracranial electrophysiological human study provides the first evidence of patients rapidly gaining real-time control over ongoing deep-brain oscillatory activity through visual neurofeedback. Moreover, neurofeedback-learnt control improves with training duration, is retained in the immediate and short-term even in the absence of neurofeedback and improves motor performance. This novel approach could enable PD patients to regain control over aberrant deep brain signalling as well as result in better motor control and, thus, complement current treatment approaches.

## Supplementary Information

Supplementary Figures.
