# Supplementary material for: Deep brain electrical neurofeedback allows Parkinson patients to control pathological oscillations and quicken movements
Source: Sci Rep. 2021 Apr 12;11:7973. doi: 10.1038/s41598-021-87031-2 (PMC8041890; doi:10.1038/s41598-021-87031-2)
Supplement: Supplementary file 1 — Supplementary Figures. [file 41598_2021_87031_MOESM1_ESM.docx]

Supplementary information

**Deep brain electrical neurofeedback allows Parkinson patients to control pathological oscillations and quicken movements**

**Authors**: Oliver Bichsel^1,2,3,4^, Lennart H. Stieglitz^3,4^, Markus F. Oertel^3,4^, Christian R. Baumann^2,4^, Roger Gassert*^1^, Lukas L. Imbach*^,2,4,5^ (*co-senior authors)

1. Rehabilitation Engineering Laboratory, Department of Health Sciences and Technology, ETH Zurich, Switzerland
2. Department of Neurology, University Hospital Zurich, University of Zurich, Switzerland
3. Department of Neurosurgery, University Hospital Zurich, University of Zurich, Switzerland
4. Clinical Neuroscience Centre, University Hospital Zurich, University of Zurich, Switzerland
5. Swiss Epilepsy Center, Klinik Lengg, Switzerland

**Corresponding Author**: Oliver Bichsel, MD, MSc; [oliver.bichsel@hest.ethz.ch](mailto:oliver.bichsel@hest.ethz.ch)

**Supplementary Figure 1: Neurofeedback experiment.** The neurofeedback experiment consisted of three parts: the pre-neurofeedback, the neurofeedback and the transfer part. There was an additional part two days later: the 2 d transfer run. In the flow-chart, the blocks that were followed by a period of 15 s of behavioural assessment are indicated by ’15 s pro-/supination’.


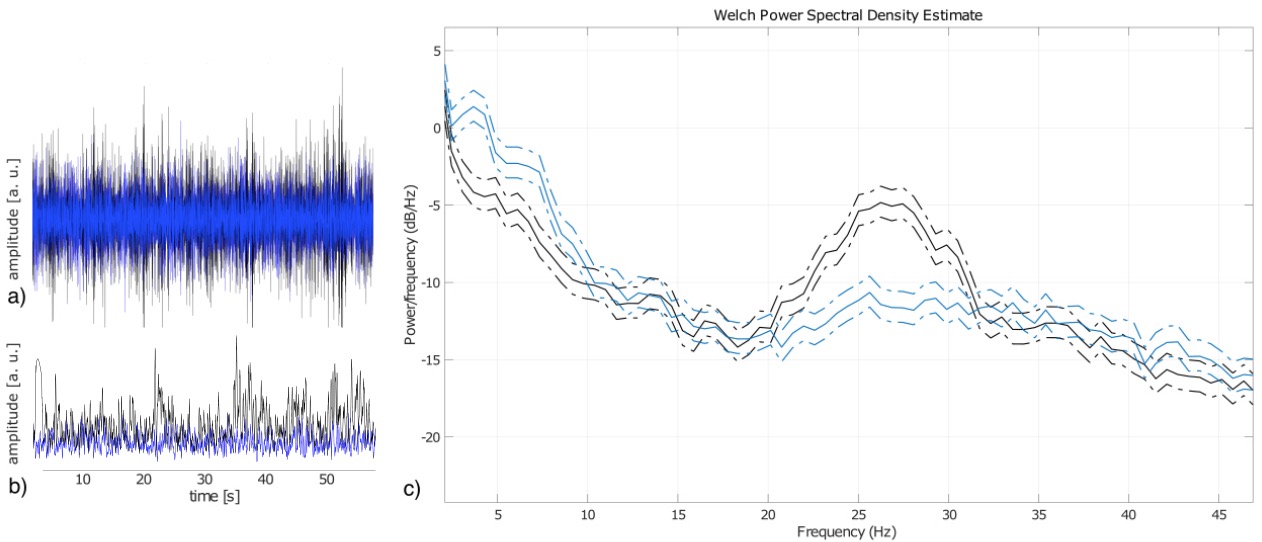


**Supplementary Figure 2: Representative beta-activity recording during neurofeedback.** These sample recordings show instantaneous beta-reduction in a patient during downregulation (blue, lower amplitude) as compared to rest (black, higher amplitude) in NF3. a) Beta-peak-filtered signal, b) 1’000 point moving average (200 ms), c) Welch power spectral density estimate with an oscillatory power reduction specific to the beta-peak (around 26 Hz) during downregulation.

**Supplementary Figure 3: Representative motor behaviour.** Filtered gyroscopic data (6^th^ order Butterworth, bandpass [0.25, 4] Hz) from the palm of a sample patient during a 15 s pro- and supination task following the NF3 and the short-term transfer blocks (blue). The first 12 s were extracted for further analysis (red).
